# Inhibition of MMP-2 and MMP-9 by Dietary Antioxidants in THP-1 Macrophages and Sera from Patients with Breast Cancer

**DOI:** 10.3390/molecules29081718

**Published:** 2024-04-10

**Authors:** Tiziana Latronico, Tania Petraglia, Carmela Sileo, Domenico Bilancia, Rocco Rossano, Grazia Maria Liuzzi

**Affiliations:** 1 Department of Biosciences, Biotechnologies and Environment, University of Bari “Aldo Moro”, 70126 Bari, Italy; tiziana.latronico@uniba.it (T.L.); graziamaria.liuzzi@uniba.it (G.M.L.); 2 Department of Sciences, University of Basilicata, 85100 Potenza, Italy; tania.petraglia@unibas.it (T.P.); carmelasileo29@gmail.com (C.S.); 3 Operating Unit, Medical Oncology, Hospital “Azienda Ospedaliera S. Carlo”, 85100 Potenza, Italy; domenicobilancia@gmail.com

**Keywords:** dietary antioxidants, matrix metalloproteinases, breast cancer, THP-1 macrophages, oxidative stress

## Abstract

Polyphenols, the main antioxidants of diet, have shown anti-inflammatory, antioxidant and anticarcinogenic activities. Here, we compared the effects of four polyphenolic compounds on ROS production and on the levels of matrix metalloproteinase (MMP)-2 and -9, which represent important pathogenetic factors of breast cancer. THP-1 differentiated macrophages were activated by LPS and simultaneously treated with different doses of a green tea extract (GTE), resveratrol (RSV), curcumin (CRC) and an olive fruit extract (oliplus). By using the 2,2-Diphenyl-1-picrylhydrazyl (DPPH) radical scavenging assay, we found that all of the tested compounds showed antioxidant activity in vitro. In addition, GTE, RSV and CRC were able to counteract ROS production induced by H_2_O_2_ in THP-1 cells. As assessed by a zymographic analysis of THP-1 supernatants and by an “in-gel zymography” of a pool of sera from patients with breast cancer, the antioxidant compounds used in this study inhibited both the activity and expression of MMP-2 and MMP-9 through different mechanisms related to their structures and to their ability to scavenge ROS. The results of this study suggest that the used antioxidants could be promising agents for the prevention and complementary treatment of breast cancer and other diseases in which MMPs play a pivotal role.

## 1. Introduction

Breast cancer stands as one of the most prevalent and life-threatening malignancies affecting women worldwide [1]. This multifaceted disease arises from the uncontrolled growth of abnormal breast cells and can manifest in various forms, making it a formidable challenge in the realm of oncology. Although it is a multifactorial pathology, it is now established that lifestyle can affect the onset and development of breast cancer. In this regard, it can be noted that with globalization, in recent years, the incidence of breast cancer has increased in developing countries where the habits of Western countries are taking over. It has been highlighted, in fact, that a sedentary lifestyle and a diet rich in saturated fatty acids and poor in fibers and vegetables affects the onset and relapses of breast cancer [2,3].

Understanding the intricate molecular mechanisms underlying breast cancer is crucial for developing effective diagnostic tools and therapeutic strategies.

It is now known that inflammation plays a key role in tumor development and progression and that these processes are driven by a complex interplay between malignant tumor cells and the surrounding nonmalignant stroma, comprising the extracellular matrix (ECM); stromal cells such as endothelial cells (ECs); fibroblasts; and infiltrating immune cells. Among infiltrated immune cells, tumor-associated macrophages (TAMs) represent the primary resident cells of the breast tumor microenvironment (TME) and are the main cells implicated in inflammatory processes [4] via their ability to secrete inflammatory factors such as pro-inflammatory cytokines and matrix metalloproteinases (MMPs) [5,6].

MMPs are part of a family of proteolytic enzymes responsible for remodeling the ECM, a dynamic network of proteins that provides structural support to tissues. MMPs are essential for normal physiological processes, like tissue repair and organ development, but their dysregulated activity has been intricately linked to cancer progression. In the context of breast cancer, MMPs have garnered significant attention due to their role in several key aspects of cancer progression, including invasion, metastasis and angiogenesis [7]. Elevated levels of specific MMPs, such as gelatinases MMP-2 and MMP-9, have been found in breast cancer tissues and are associated with a more aggressive tumor phenotype [8]. MMPs can promote cancer cell invasion into surrounding tissues and facilitate the establishment of metastases [9]. Additionally, MMPs can indirectly affect the tumor microenvironment by altering the ECM composition, thus contributing to the remodeling process.

Although it was initially assumed that MMPs present in the tumor microenvironment were produced by tumor cells, in situ hybridization techniques have demonstrated that MMPs are predominantly produced by adjacent host stromal and inflammatory cells in response to factors released by tumors [10].

An important role in breast cancer initiation and progression is also played by oxidative stress. Oxidative stress, generated by an imbalance between reactive oxygen species (ROS) and endogenous antioxidants, can contribute to DNA damage, genetic mutations and alterations to cellular signaling pathways, which can lead to the transformation of normal breast cells into cancerous cells. Additionally, oxidative stress may promote the survival and growth of breast cancer cells and contribute to resistance to chemotherapy and radiation therapy [11].

It is now common knowledge that there is an interplay between oxidative stress and MMPs. Reactive oxygen species have been implicated in MMP-9 zymogen activation [12]. In addition, oxidative stress can lead to the activation of signaling pathways that regulate MMP expression. For example, the nuclear factor-kappa B (NF-κB) pathway, which is activated in response to oxidative stress, can stimulate the expression of certain MMPs [11].

Based on these considerations, MMPs represent attractive therapeutic targets that are susceptible to antioxidant modulation. Therefore, compounds capable of inhibiting MMPs and, at the same time, neutralizing ROS, can act as powerful bullets in the fight against breast cancer.

Polyphenols are ubiquitous organic molecules present in vegetables, fruits, coffee, tea, cereals and wine. Together with vitamins, they represent the main antioxidants of diet. Polyphenols are characterized by the presence of hydroxylated phenolic groups organized in more or less complex structures. Based on the structural elements that bind these phenolic rings, polyphenols are divided into two categories, namely flavonoids (flavones, flavanones, flavanols, flavonols, isoflavones and anthocyanins) and non-flavonoids (phenolic acids, stilbenes, coumarins, curcumin, tannins and lignans) [13].

**Figure 1:**
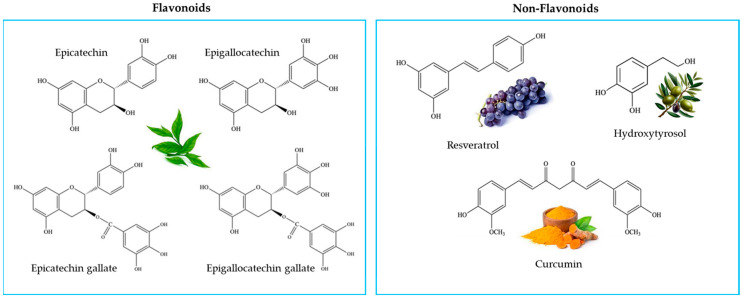
The structures of the different polyphenols used in this study showing the nature of their chemical groups.

The aim of this study was to evaluate the antioxidant activity of four different dietary polyphenolic compounds and their ability to inhibit ROS production in THP-1 macrophages and the activity of MMP-2 and MMP-9 in both THP-1 macrophages and in sera from patients with breast cancer. For our experiments, as a member of a flavonoids group, we chose a green tea extract (GTE) containing high concentrations of the flavanols catechin, epicathechin, epigallocatechin, epicatechin gallate and epigallocatechin gallate, whereas the other antioxidants were represented by three non-flavonoids: resveratrol (a stilbene), curcumin and tyrosol/hydroxytyrosol. The latter are the main antioxidants present in an olive fruit extract (oliplus) (Figure 1).

The results suggest that the polyphenols used in this study exert antioxidant activity at low concentrations and inhibit MMP-2 and MMP-9 activity with different mechanisms of action possibly related to their different structures. On these grounds, a diet rich in polyphenols characterized by different chemical structures may represent a winning strategy for the inhibition of key factors, such as MMPs, involved in breast cancer.

## 2. Results

### 2.1. Antioxidant Activity In Vitro

**Figure 2:**
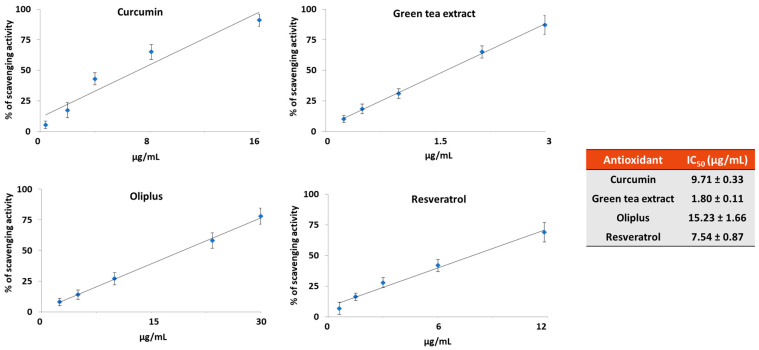
The scavenging activities of the dietary antioxidants. The graphs represent the scavenging activities of the antioxidants against 2,2-Diphenyl-1-picrylhydrazyl (DPPH). The results are expressed as the IC_50_ values that represent the concentration of antioxidant necessary to scavenge 50% of free radicals. The data are shown as the mean values ± S.D. of three replicates.

Figure 2 shows the free radical scavenging activity of the four dietary antioxidant compounds used, as detected by the 2,2-Diphenyl-1-picrylhydrazyl (DPPH) radical scavenging assay. Among the studied compounds, the highest antioxidant activity was observed for the green tea extract (GTE) (1.80 ± 0.11 µg/mL), followed by resveratrol (RSV) (7.54 ± 0.87 µg/mL) and curcumin (CRC) (9.71 ± 0.33 µg/mL), whereas the lowest antioxidant activity (15.23 ± 1.66 µg/mL) was measured in the extract derived from olive fruits (OLI).

### 2.2. Effect of Antioxidant Compounds on THP-1 Macrophage Viability

**Figure 3:**
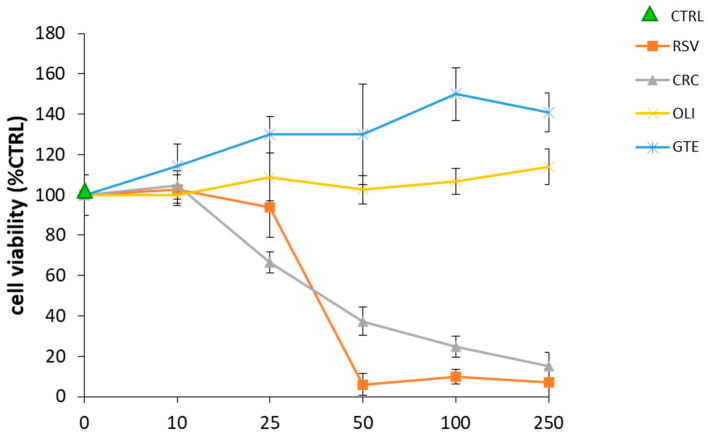
The cell viability of the THP-1 macrophages treated with the antioxidant compounds. The THP-1 macrophages were treated for 20 h with resveratrol (RSV), oliplus (OLI), green tea extract (GTE) or curcumin (CRC) at the indicated concentrations and then subjected to the MTT assay. The control (CTRL) was represented from untreated cells in serum-free RPMI. The graphs represent the dose–response curves of cell viability, expressed as the percentage of cell survival in comparison to the control, which was set at 100%. The horizontal dashed line, set at 60%, indicates the threshold of cell viability. Concentrations of antioxidant compounds that yielded cell viability values <60% of the control were considered toxic doses. The values are shown as the mean ± SD of *n* = 3 experiments performed on different cell populations.

Preliminary experiments were performed to identify the concentrations of the antioxidant compounds that were not toxic for cells, a basic assumption to exclude that the effects observed were the consequence of cellular suffering. To this purpose, differentiated THP-1 macrophages were treated for 20 h with the antioxidants at concentrations ranging between 10 and 250 μg/mL, as described in the Materials and Methods Section. As shown in Figure 3, among the investigated compounds, GTE and OLI were the least toxic since they did not show any toxicity at any of the concentrations tested. By contrast, CRC and RSV were toxic to cells at concentrations higher than 25 μg/mL. No difference in cell viability was observed in the cells activated with LPS and treated with the antioxidants. 

### 2.3. Effect of Antioxidant Compounds on MMP-2 and MMP-9 Released from LPS-Activated THP-1 Macrophages

**Figure 4:**
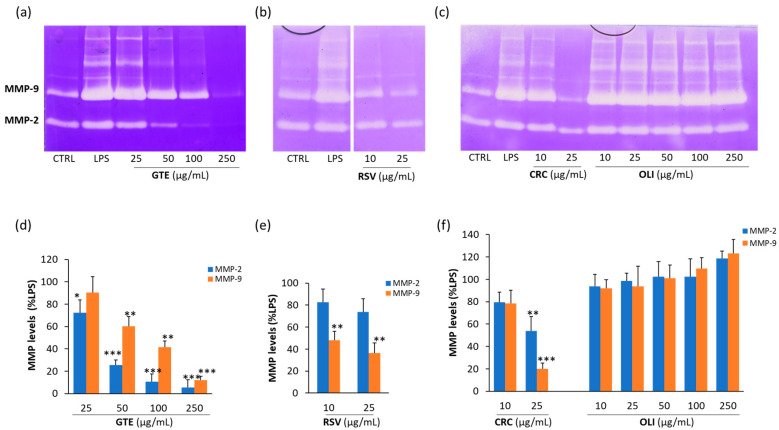
Effect of green tea extract (GTE), resveratrol (RSV), curcumin (CRC) and oliplus (OLI) on MMP-2 and MMP-9 levels in LPS-activated THP-1 macrophages. (**a**–**c**) Representative zymographic gels in (**a**–**c**) show MMP-2 and MMP-9 levels in culture supernatants from THP-1 macrophages activated with LPS (10 μg/mL) and simultaneously treated for 20 h with GTE, RSV, CRC or OLI at indicated concentrations. Negative and positive controls were obtained from unstimulated and untreated cells in serum-free medium (CTRL) and LPS-activated cells (LPS), respectively. Histograms in (**d**–**f**) represent mean ± SD of *n* = 3 experiments performed on different cell populations. Results are expressed as percentage of MMP levels in comparison to LPS, calculated after scanning densitometry and computerized analysis of gels. Asterisks represent values statistically different from positive control (one-way ANOVA followed by Dunnet’s post hoc test; * *p* < 0.05, ** *p* < 0.01, *** *p* < 0.001).

As shown in the representative zymograms reported in Figure 4a–c, the treatment of THP-1 macrophages with LPS increased the levels of both MMP-2 and MMP-9. By contrast, the co-treatment with GTE, RSV or CRC induced a dose-dependent inhibition of MMP-2 and MMP-9. Differently, OLI had no inhibitory effects on the two proteinases. The statistical analysis of data (Figure 4d–f) evidenced that CRC was the most potent antioxidant among those used, since at the concentration of 25 µg/mL, it was able to achieve the highest inhibition of both the MMP-2 and MMP-9 levels (45 and 80%, respectively) compared to the other antioxidants at the same concentration. However, RSV at the lowest concentration used (10 μg/mL) was the only antioxidant capable of inducing a statistically significant inhibition of MMP-9 (approximately 50%). At the maximum non-toxic concentration of 250 μg/mL, GTE completely inhibited the activity of both MMP-2 and MMP-9.

### 2.4. Effect of Antioxidant Compounds on ROS Production in THP-1 Macrophages Treated with Hydrogen Peroxide

To evaluate the potential antioxidant activity of the studied compounds, differentiated THP-1 macrophages were pretreated with GTE, RSV, OLI or CRC at concentrations ranging between 5 and 25 μg/mL, and then activated with hydrogen peroxide (H_2_O_2_) as described in the Materials and Methods Section.

**Figure 5:**
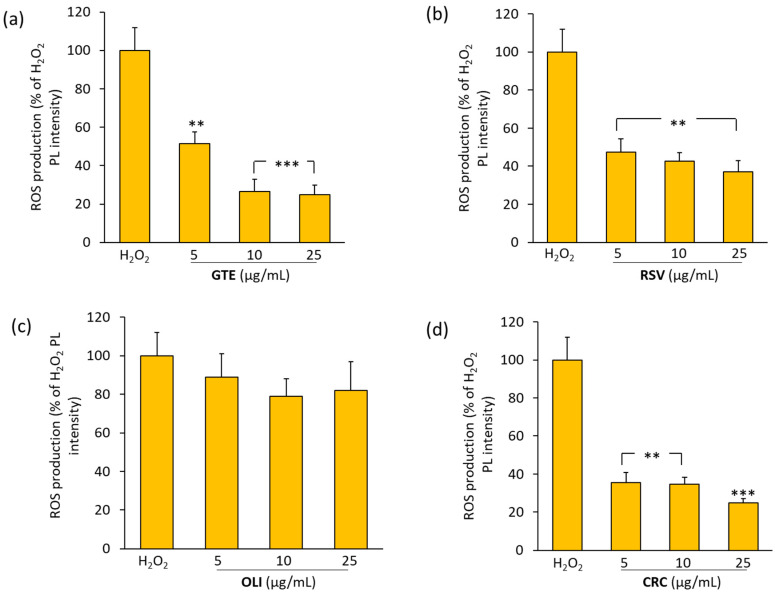
The effects of green tea extract (GTE), resveratrol (RSV), oliplus (OLI) and curcumin (CRC) on ROS in THP-1 macrophages. ROS production, assayed by measuring the changes in the fluorescent signal of 2′,7′-dichlorofluorescein (DCFA), was expressed as the percentage (%) of photoluminescence (PL) intensity in comparison to the positive control (100%), represented by cells treated with 100 μM H_2_O_2_. The histograms (**a**–**d**) represent the mean values ± SD of *n* = 3 experiments performed on different cell populations. A statistically significant decrease in comparison to H_2_O_2_ is indicated by asterisks (one-way ANOVA followed by Dunnet’s post hoc test; ** *p* < 0.01 and *** *p* < 0.001).

As shown in Figure 5 GTE, RSV and CRC showed a statistically significant ability to counteract the H_2_O_2_-induced ROS production. Among the compounds tested, CRC and RSV were the most effective; at the dose of 5 μg/mL, they were able to induce reductions of about 65 and 50%, respectively, in the ROS levels. However, at the concentration of 25 μg/mL, CRC and GTE were the most efficacious compounds in counteracting H_2_O_2_-induced ROS production (75%). No statistically significant inhibition of ROS was observed by OLI at any of the doses used.

### 2.5. “In-Gel” Inhibition of MMP-2 and MMP-9 Activity by Antioxidant Compounds

**Figure 6:**
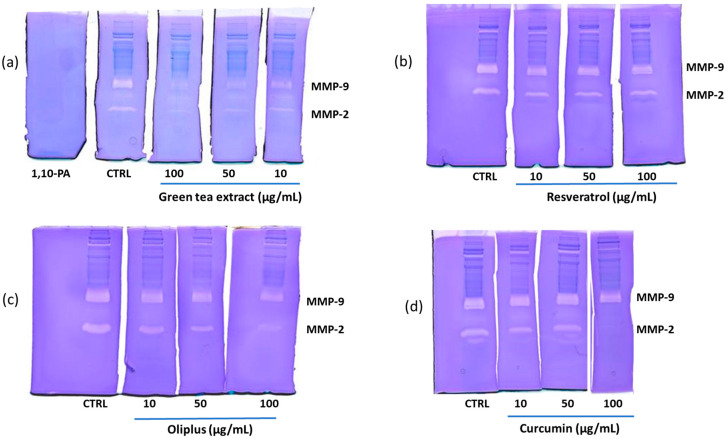
The “in-gel” inhibition of MMP-2 and MMP-9 activity by the antioxidant compounds. A pool of sera obtained from patients affected by breast cancer was applied to 1D gelatin zymography. After electrophoresis, gels were cut in lanes, and each lane was incubated individually in the presence of the following single antioxidants at different concentrations: green tea extract (**a**), resveratrol (**b**), oliplus (**c**) and curcumin (**d**), respectively.

Figure 6 shows the results on the ability of antioxidants to inhibit the “in-gel” activity of MMP-2 and MMP-9 in a pool of sera obtained from patients affected by breast cancer. In Figure 6a–d, in the lanes corresponding to the control (CTRL, lanes of sera incubated in the absence of antioxidants), two main bands of digestion corresponding to MMP-9 and MMP-2 are observed. The incubation with 1,10 phenanthroline (1,10-PA), a specific inhibitor of metalloproteinases, completely inhibited the activity of both MMP-2 and MMP-9 (gel a). Treatment with GTE (gel a) determined an inhibition for both gelatinases in a concentration-dependent manner. As shown in gel b, RSV induced a slight inhibition of MMP-2 activity, whereas it did not exert any inhibitory activity on MMP-9 at any of the concentrations tested. In the lanes incubated with OLI (gel c), a dose-dependent reduction in MMP-2 activity was evident, whereas MMP-9 was inhibited at a low extent only at the highest concentrations of 50 and 100 µg/mL. Finally, CRC (gel d) induced a strong inhibition of MMP-2 at the highest concentration of 100 µg/mL and did not inhibit MMP-9.

The percentages of inhibition of MMP-2 and MMP-9, calculated in comparison with CTRL, after the densitometric analysis of gels, are shown in Table 1.

## 3. Discussion

In the past two decades, numerous studies have been directed towards the functional role of diet for the prevention and complementary treatment of numerous pathologies including tumors and cardiovascular and neurodegenerative diseases [14]. A common hallmark of all of these multifactorial diseases is represented by inflammation. To understand how nutrition can improve the course of these pathologies, it is important to identify the molecular mechanisms through which food molecules are able to dampen inflammatory processes by intervening on cellular targets.

Among the molecules introduced by diet, polyphenols are the most studied compounds for their multiple biological functions. Their biological effects are mainly due to their anti-inflammatory, immunomodulatory, anti-angiogenic, neuroprotective, antioxidant and radical scavenging properties that might be beneficial for the prevention and treatment of different diseases, including breast cancer [15,16,17].

In this paper, we compared the effects of two purified polyphenols (CRC and RSV) and two extracts rich in polyphenols (GTE and OLI), which are present in numerous vegetables that are commonly consumed, on the production of ROS and on the levels of MMP-2 and MMP-9 in an experimental model represented by THP-1 cells differentiated in macrophages and activated with LPS.

Macrophages, key components of the immune system, play a multifaceted role in the context of cancer, particularly breast cancer [18]. In the tumor microenvironment, tumor-associated macrophages (TAMs) often adopt distinct phenotypes, ranging from pro-inflammatory (M1) to anti-inflammatory (M2), influenced by signals from the surrounding milieu. The crosstalk between breast cancer cells and inflammatory macrophages not only influence the tumor microenvironment but also impact various aspects of cancer biology, such as proliferation, invasion, angiogenesis and metastasis.

TAMs are implicated in the generation of ROS, contributing to oxidative stress within the tumor microenvironment, which, in turn, can promote tumor progression through various mechanisms, including genomic instability, DNA damage and the activation of signaling pathways promoting the release of pro-inflammatory molecules [19].

THP-1 macrophages represent a good model of study and are already used by other authors because, through their activation with stimuli such as LPS, they can be differentiated into a pro-inflammatory M1-like phenotype that is capable of secreting pro-inflammatory factors including cytokines, ROS and enzymes such as MMPs [4].

In this study, we chose MMPs as targets of the effects of the studied polyphenols since these enzymes play a key role in cancer cell invasion by digesting the ECM, supporting cancer cell growth and tumor metastasis [20,21]. In this contest, numerous attempts have been made to generate drugs that are capable of inhibiting MMPs, mainly with the aim of blocking the invasion and metastasis of tumor cells, but the results of clinical trials have proved disappointing, with unwanted side effects and a lack of effectiveness [22]. To date, there are no MMP inhibitors available at the clinical level for the treatment of cancer, or those available are not therapeutically useful due to the lack of specificity [23]. As reported by Vandooren et al. [24], there is a persistent lack of knowledge on the complexity of MMP biology, which hinders the development of safe and effective MMP-targeted drugs. On these bases, the use of natural compounds as dietary antioxidants that are able to inhibit MMPs with different mechanisms of action may represent an interesting challenge for research, considering the role that ROS play in the activation of MMPs [25].

Several phytochemicals have been found to act as direct inhibitors of MMP activation or as modulators of signaling pathways associated with MMP expression [20,26,27]. In this regard, numerous studies have demonstrated the ability of polyphenols to inhibit MMP activity and expression [28,29,30,31,32].

By using the DPPH radical scavenging assay, we measured the in vitro antioxidant activity of RSV, CRC, OLI and GTE and found that all of the tested compounds are good antioxidants which act at low concentrations. In accordance with the data reported in the literature, we found different antioxidant capacities for the studied compounds, which were comparable to those found by other authors using the same DPPH assay [33,34,35,36]. As already reported, the different antioxidant capacities of the studied polyphenols could be attributed to their different molecular structures [30]. Indeed, the antioxidant power of polyphenols may depend on the number of phenolic rings, the number and positions of hydroxyl groups and the double bonds present in the molecule.

The results of this study indicate that the used polyphenols are able to inhibit MMP-2 and MMP-9 in relation to their antioxidant power. Among the antioxidants used in this study, RSV, CRC and GTE were able to counteract the increases in the MMP-9 and MMP-2 levels with a different inhibitory capacity, whereas OLI, which was the compound with the lowest antioxidant power, was not able to significantly inhibit the levels of MMP-2 and MMP-9 released in THP-1 supernatants. The lack of inhibition of MMPs by OLI could depend on the doses used, the composition of the extract and the cell population tested. In fact, in another work in which OLI was tested on a different cell population and at a higher concentration, this antioxidant significantly inhibited MMP-2 and MMP-9 levels [30]. Similarly, an inhibitory effect on MMP-9 was observed by other authors using a different extract of olive oil or hydroxytyrosol [32,37].

The cytotoxicity test performed on cells treated with the antioxidants indicated that the used compounds were not toxic for cells at the inhibitory doses, and therefore, the observed changes in the levels of MMP-9 and MMP-2 in LPS-activated THP-1 supernatants were due to a real inhibitory effect on gelatinases.

**Figure 7:**
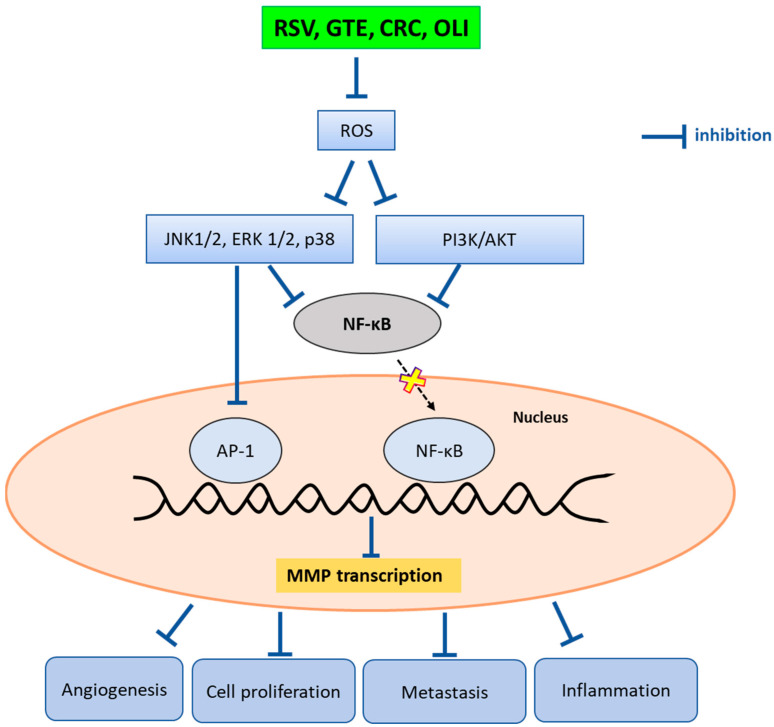
Antioxidant-mediated downregulation of MMP gene expression via ROS-triggered signal transduction pathways.

One important mechanism by which polyphenols exert their anti-MMP activities is likely to be via the downregulation of ROS. Numerous studies have demonstrated a strict interplay between MMP levels and ROS production [38]. ROS activate signaling pathways that are involved in the transcriptional activation of the gene that promotes MMP expression (Figure 7) [39].

In this work, we tested the ability of the antioxidants used to block the production of ROS in THP-1 cells activated with a non-specific pro-oxidant stimulus represented by hydrogen peroxide. We tested concentrations between 5 and 25 µg/mL to compare the ROS scavenging capacity between all of the antioxidants used. The results obtained evidence that GTE, RSV and CRC showed a protective effect in counteracting the oxidative stress induced in THP-1 cells by hydrogen peroxide. The antioxidant that was less effective in blocking the production of ROS was OLI. This result seems to confirm our hypothesis, according to which the ability to block the production of ROS, resulting in the inhibition of MMP-2 and MMP-9 levels in THP-1 cells, could be related to the antioxidant power of the compounds tested.

According to the results obtained from the analysis of the THP-1 supernatants and from the “in-gel” zymography of a pool of sera from patients with breast cancer, we can hypothesize that, as suggested by other authors, the antioxidant compounds used in this study can inhibit MMPs through different mechanisms, involving the inhibition of both MMP activity and expression, with the latter occurring through the downregulation of different signaling pathways involved in MMP-2 and MMP-9 gene transcription (Figure 7). At the molecular level, polyphenols may downregulate the ROS-induced activation of ERK 1/2, p38 and JNK, as well as PI3K/AKT, preventing the nuclear translocation of NF-kB and the downstream activation of the transcription factor AP-1. The consequent downregulation of MMP gene transcription results in the inhibition of tumor cell growth, metastasis, angiogenesis and inflammation [22,40,41]. As a result of our experiments, RSV inhibited MMP-9 in THP-1 supernatants but not in “in-gel” zymography, suggesting that this antioxidant only exerted its inhibitory action on the expression of the enzyme. This result is consistent with previous studies showing the ability of RSV to downregulate MMP-9 expression by inhibiting the activation of the transcription factors NF-kB and AP-1 [30,40].

Differently, we found that GTE was able to inhibit, in a dose-dependent manner, both the activity and expression of MMP-2 and MMP-9 as assessed by the “in-gel” zymography and by the analysis of the THP-1 supernatants, respectively.

By contrast, CRC inhibited MMP-2 and MMP-9 with different mechanisms. In fact, the inhibition of MMP-9 seems to be directed exclusively on the expression of the enzyme, as demonstrated by the analysis of the THP-1 supernatants. Conversely, MMP-2 expression and activity levels were both inhibited. In the latter case, CRC, at the highest concentration used, inhibited MMP-2 with an efficiency comparable to that of 1,10 phenanthroline, a specific inhibitor of MMPs. However, it must be considered that, due to the cytotoxicity of CRC on THP-1 cells, it was not possible to compare its inhibitory effect at concentrations higher than 25 μg/mL in the two systems used.

One of the possible mechanisms to explain the “in-gel” inhibition of MMPs by polyphenols could be their ability to chelate metals [42], including zinc [43,44], the indispensable cofactor for the activity of MMP-2 and MMP-9. Polyphenols are excellent metal chelators; their chelating capacities are closely linked to the presence of catechol groups and the combination of hydroxyl and carbonyl groups from which the metal binding sites originate. Polyphenols with galloyl or catechol groups are more potent metal chelators than those without these groups [45]. Lakey-Beitia et al. [46] proposed that polyphenols could be classified into three groups: a group with only one binding site for metals that includes most polyphenols, such as curcuminoids, lignans, stillbenes, isoflavonoids, flavanols and anthocyanins; a group with two binding sites, including flavones and flavanones; and finally, a group with three binding sites, which includes flavonols, flavanols and tannins. All of these considerations could explain the reason why, in our experiments, GTE is the most active “in-gel” inhibitor of MMPs even at the lowest concentrations. However, as reported by Suzuki et al. [40], another important mechanism to explain the inhibitory activity of polyphenols towards MMPs might be their direct binding to MMPs, as demonstrated by several molecular docking studies [47,48,49,50].

On these bases, the natural MMP inhibitors tested in this study could be used to plan alternative therapeutic strategies for the treatment of cancer in synergy with conventional therapies.

## 4. Materials and Methods

### 4.1. Chemicals and Reagents

Gelatin, DNase, Roswell Park Memorial Institute (RPMI) 1640 medium, Roswell Park Memorial Institute (RPMI) 1640 medium without Phenol Red, fetal bovine serum (FBS), penicillin and streptomycin were provided by GIBCO (Paisley, Scotland). Trypan Blue, 3-(4,5-dimethylthiazol-2-yl)-2.5-diphenyltetrazolium bromide (MTT), 1,10 phenanthroline (PA), phorbol 12-myristate 13-acetate (PMA), lipopolysaccharide (LPS), hydrogen peroxide (H_2_O_2_) and 2,2-diphenyl-1-picrylhydraziyl radical (DPPH) were provided by Sigma (St. Louis, MO, USA). 2′,7′-dichlorofluorescein diacetate (DCFH-DA) was purchased from Calbiochem (Milano, Italy). Standard proteins and R-250 Coomassie Brilliant Blue were from Bio-Rad (Hercules, CA, USA). Human monocytic leukemia cell line (THP-1 ATCC^®^ TIB-202™) was obtained from American Type Culture Collection (ATCC), Manassas, VI, USA.

### 4.2. Antioxidants

The antioxidant samples, namely resveratrol (99.7%); oliplus (total polyphenols: 45.5%); green tea extract (total polyphenols: 50%) and curcumin (96.6%), were purchased from Nutraceutica s.r.l. (Monterenzio, (BO), Italy). All of the antioxidants, except for green tea extract (prepared in 40% ethanol in water solution), were solubilized with 80% ethanol in water solution.

### 4.3. DPPH Radical Scavenging Activity

2,2-Diphenyl-1-picrylhydraziyl radical (DPPH) is commonly employed to measure the capability of antioxidant compounds to scavenge free radicals or serve as hydrogen providers. DPPH is a deep purple nitrogenous organic radical that absorbs at 517 nm. When an antioxidant compound reacts with the DPPH radical, the reduced molecular form is generated; thus, the absorbance decreases proportionally and can be spectrophotometrically monitored over time at 517 nm. The capacity of antioxidants to scavenge DPPH was evaluated as reported by Petraglia et al. [51]. Briefly, 0.2 mL of antioxidant samples at different concentrations was added to 0.8 mL of 0.2 mM DPPH in ethanol. After 30 min of incubation at room temperature in the dark, absorbance was measured at 517 nm. Results were expressed as percentage of DPPH radical scavenging using the following equation: (%) = [(Abs control − Abs sample)/Abs control] × 100. Antioxidant activity was expressed as IC_50_, representing the sample concentration (µg/mL) required to scavenge 50% of DPPH free radicals.

### 4.4. THP-1 Differentiation

THP-1 monocytes were maintained in T-75 flasks (75 cm^2^) in Roswell Park Memorial Institute (RPMI) 1640 Medium supplemented with 100 U/mL penicillin, 100 μg/mL streptomycin, 10% FBS at 37 °C and 5% CO_2_. For differentiation into macrophages, THP-1 monocytes were plated at a density of 1.5 × 10^5^ in 96-well plates and incubated for 24 h with 1 μM phorbol 12-myristate 13-acetate (PMA). After incubation for another 24 h in RPMI medium, THP-1, differentiated in macrophages, was used for the different assays.

### 4.5. Cell Viability

To assess the biocompatibility of antioxidant compounds, THP-1 macrophages plated in 96-well plates in serum-free medium were treated with resveratrol (RSV), oliplus (OLI), green tea extract (GTE) or curcumin (CRC) at the final concentrations of 10, 25, 50, 100 and 250 μg/mL. After incubation for 20 h at 37 °C and 5% CO_2_, the culture medium was removed, and the cell viability was evaluated by MTT [3-(4,5-dimethylthiazol-2-yl)-2,5-diphenyl tetrazolium bromide] assay as previously reported [52].

### 4.6. Treatment of LPS-Activated THP-1 Macrophages with Antioxidant Compounds

Differentiated THP-1 macrophages, plated in 96-well microplates in serum-free medium, were stimulated with 10 μg/mL of LPS and simultaneously treated with different concentrations of RSV, OLI, GTE and CRC. THP-1 macrophages in serum-free RPMI 1640 medium and LPS-activated THP-1 macrophages represented negative and positive controls, respectively. After 20 h of incubation at 37 °C and 5% CO_2_, the culture medium was collected and stored at −80 °C until analysis, whereas the cells were subjected to MTT assay to assess cell viability.

### 4.7. Reactive Oxygen Species Detection

To evaluate the potential antioxidant activity of the studied compounds, differentiated THP-1 macrophages were plated in 96-well microplates and pretreated with GTE, OLI, RSV and CRC at concentrations in the range of 5–25 µg/mL in RPMI without phenol R = red. After 1 h of incubation, cells were stimulated for 1 h with 100 μM of H_2_O_2_ in the presence of antioxidant compounds, and the detection of ROS was performed by loading cells with 10 μM of 2′,7′-dichlorofluorescein diacetate (DCFH-DA) in phenol-red-free RPMI, as previously reported [53]. After incubation for 30 min at 37 °C, the culture medium was removed, and cells were rinsed twice with PBS. Cells were resuspended in phenol-red-free RPMI, and a spectrofluorometric analysis was performed at 485 nm excitation/525 nm emission using a multi plate reader (Cytation 3, BioTek, Winooski, VT, USA). Negative control was represented by cells treated only with DCFH-DA in the same experimental conditions. H_2_O_2_-stimulated THP-1 macrophages represented the positive control (H_2_O_2_). The ability of the antioxidant compounds to inhibit ROS production was expressed as relative percentage of photoluminescence intensity (PLI) in comparison to the positive control.

### 4.8. Detection of MMP-2 and MMP-9 by Zymography

The MMP-2 and MMP-9 levels in cell supernatants were detected by zymographic analysis as reported by Latronico et al. [52]. Briefly, 50 μL of supernatants, containing about 10 μg of total proteins, was precipitated with 1 mL of ice-cold acetone. After incubation for 1 h at −20 °C and centrifugation at 13,000× *g* at 4 °C, dry pellets were solubilized with 15 μL of Laemmli sample buffer without β-mercaptoethanol. Samples were run in a 7.5% polyacrylamide gel copolymerized with 0.1% (*wt*/*v*) gelatin. After the electrophoretic run at 120 V, gels were rinsed twice with 2.5% Triton X-100/10 mM CaCl_2_ in 50 mM Tris–HCl, pH 7.4 (washing buffer) and incubated for 24 h at 37 °C in 1% Triton X-100/50 mM Tris–HCl/10 mM CaCl_2_, pH 7.4 (incubation buffer). After staining and destaining of gels, MMP-2 and MMP-9 levels were visualized as a clear band of digestion on a blue background of the gel and were quantified by computerized densitometric image analysis using Image LabTM Software, Version 5.2 (Bio-Rad Laboratories, Hercules, CA, USA). Gelatinase levels were expressed as percentage of positive control (LPS-activated cells).

### 4.9. Determination of Inhibitory Capacity (In-Gel Inhibition) of Dietary Antioxidant on Gelatinases Present in Pool of Sera from Patients with Breast Cancer 

The effects of natural antioxidants on the activity of gelatinases present in a pool of sera from patients with breast cancer were evaluated by one-dimensional zymography on polyacrylamide gel copolymerized with gelatin as already reported [54]. The serum samples were provided by the Medical Oncology Unit of the S. Carlo Hospital in Potenza (ethical committee authorization no. 74/2021). To perform the inhibition tests, 1.5 μL of aliquots of the serum pool was analyzed by “in-gel” zymography. After reactivation of the proteases, the various lanes were cut from the gels and incubated individually (for 16 h at 37 °C) in incubation buffer containing various antioxidants at the concentrations of 10, 50 and 100 µg/mL. 1,10-phenanthroline (1,10 PA) was used as a positive control. After staining and destaining of gels, MMP-2 and MMP-9 activity was detected as reported in Section 4.8.

## 5. Conclusions

The results obtained in this study confirmed the role of some polyphenols extracted from natural matrices as precious allies for our health, since they act as inhibitors of important enzymes involved in pathological events, such as MMPs. It is therefore reasonable to consider polyphenols as potential reservoirs of innovative therapeutic solutions for human health. However, the changes in their structure occurring during metabolic processes could influence their inhibitory action in vivo.

To maximize the anticancer effects of polyphenols, different drug delivery nanosystems made with biocompatible materials could be used to increase their bioavailability and bioaccessibility. The main advantages derived from the encapsulation of polyphenols in biocompatible nanomaterials are represented by the increases in their stability and solubility, protection from degradation and the possibility of targeting them towards specific cells or tissues.

In conclusion, the results of this study represent a good starting point for more in-depth future studies aimed at understanding the mechanisms underlying inhibition by polyphenols, as well as the biotransformations that these compounds undergo in vivo.

## Figures and Tables

**Table 1:** In-gel inhibition of MMP-2 and MMP-9 activity by antioxidants.

	Inhibition (%)	
	MMP-2	MMP-9
1,10 PA	100	100
CTRL	0	0
Antioxidant (μg/mL)		
Green tea extract		
10	50.4 ± 3.7	56.4 ± 2.1
50	65.7 ± 2.5	53.6 ± 5.5
100	85.8 ± 6.1	89.9 ± 2.8
Resveratrol		
10	20.1 ± 1.2	3.2 ± 0.2
50	22.6 ± 1.9	2.6 ± 0.5
100	21.4 ± 1.1	2.1 ± 0.4
Oliplus		
10	34.9 ± 2.9	10.3 ± 2.3
50	59.0 ± 8.3	15.4 ± 3.3
100	74.5 ± 3.4	28.4 ± 4.1
Curcumin		
10	20.2 ± 1.3	1.3 ± 0.2
50	18.6 ± 6.3	2.2 ± 0.8
100	100	18.2 ± 3.7

CTRL: control (MMPs incubated in the absence of antioxidants); 1,10 PA: 1,10 phenanthroline, specific inhibitor of MMPs. Values represent percentage of inhibition in comparison to CTRL, calculated as mean ± SD of three independent experiments.

## Data Availability

Data are contained within the article.
